# Update on Vaccine-Derived Poliovirus Outbreaks — Worldwide, January 2023–June 2024

**DOI:** 10.15585/mmwr.mm7341a1

**Published:** 2024-10-17

**Authors:** Apophia Namageyo-Funa, Sharon A. Greene, Elizabeth Henderson, Mohamed A. Traoré, Shahzad Shaukat, John Paul Bigouette, Jaume Jorba, Eric Wiesen, Omotayo Bolu, Ousmane M. Diop, Cara C. Burns, Steven G.F. Wassilak

**Affiliations:** ^1^Global Immunization Division, Center for Global Health, CDC; ^2^Division of Viral Diseases, National Center for Immunization and Respiratory Diseases, CDC; ^3^Polio Eradication Department, World Health Organization, Geneva, Switzerland.

**Summary**

**What is already known about this topic?**

Circulating vaccine-derived polioviruses (cVDPVs) can emerge and cause paralysis in areas with low population poliovirus immunity. Since 2017, large cVDPV type 2 (cVDPV2) outbreaks have occurred, primarily in Africa.

**What is added by this report?**

During January 2023–June 2024, 74 cVDPV outbreaks (672 confirmed polio cases) were detected in 39 countries or areas. Annual cVDPV type 1 case counts declined markedly compared with those during 2022. Despite a small decline in reported cVDPV2 cases compared with those reported during 2022, the number of countries or areas reporting outbreaks remained high.

**What are the implications for public health practice?**

To achieve the Global Polio Eradication Initiative’s goal of interrupting cVDPV transmission by 2026, outbreak responses must be timely and overcome barriers to reaching children who are missed by routine and supplementary immunization activities.

## Abstract

Circulating vaccine-derived polioviruses (cVDPVs) can emerge and lead to outbreaks of paralytic polio as well as asymptomatic transmission in communities with a high percentage of undervaccinated children. Using data from the World Health Organization Polio Information System and Global Polio Laboratory Network, this report describes global polio outbreaks due to cVDPVs during January 2023–June 2024 and updates previous reports. During the reporting period, 74 cVDPV outbreaks were detected in 39 countries or areas (countries), predominantly in Africa. Among these 74 cVDPV outbreaks, 47 (64%) were new outbreaks, detected in 30 (77%) of the 39 countries. Three countries reported cVDPV type 1 (cVDPV1) outbreaks and 38 countries reported cVDPV type 2 (cVDPV2) outbreaks; two of these countries reported cocirculating cVDPV1 and cVDPV2. In the 38 countries with cVDPV2 transmission, 70 distinct outbreaks were reported. In 15 countries, cVDPV transmission has lasted >1 year into 2024. In Nigeria and Somalia, both countries with security-compromised areas, persistent cVDPV2 transmission has spread to neighboring countries. Delayed implementation of outbreak response campaigns and low-quality campaigns have resulted in further international spread. Countries can control cVDPV outbreaks with timely allocation of resources to implement prompt, high-quality responses after outbreak confirmation. Stopping all cVDPV transmission requires effectively increasing population immunity by overcoming barriers to reaching children.

## Introduction

Live, attenuated oral poliovirus vaccine (OPV) induces long-term protection against paralytic disease, and limits virus shedding in vaccinated persons with infection (*1*). Circulating vaccine-derived poliovirus (cVDPVs)* outbreaks occur when OPV-related strains undergo prolonged circulation in communities with very low immunity against polioviruses, and the genetically reverted virus has regained neurovirulence (vaccine-derived poliovirus [VDPV] emergence) (*2*,*3*). After declaration of wild poliovirus type 2 eradication in 2015, and in an effort to lower the risk for cVDPV type 2 (cVDPV2) outbreaks, immunization programs in countries using OPV switched from using trivalent OPV (tOPV) (containing types 1, 2, and 3 Sabin strains) in routine and supplementary immunization activities (SIAs) to bivalent OPV (bOPV) (containing types 1 and 3 Sabin strains) (*4*); bOPV is used for cVDPV type 1 (cVDPV1) outbreak response. After the switch from tOPV to bOPV, monovalent type 2 OPV (mOPV2) (Sabin-strain type 2) was reserved for cVDPV2 outbreak responses. Since 2021, novel oral poliovirus vaccine type 2 (nOPV2), a more genetically stable vaccine with reduced risk for reversion to neurovirulence than Sabin-strain OPV2, has been the recommended vaccine for cVDPV2 outbreak response (*5*). However, nOPV2 supply has been periodically restricted because of manufacturing delays, including during a period in early 2024. Despite the goal of permanent cessation of cVDPV2 transmission by switching from tOPV to bOPV, new cVDPV2 polio outbreaks continue to be reported in multiple countries (*5*,*6*). This report describes global polio outbreaks due to cVDPVs during January 2023–June 2024 and updates previous reports.

## Methods

### Data Sources

The surveillance and virologic data on cVDPV outbreaks in this report (as of September 18, 2024)^†^ were gathered from the World Health Organization (WHO) Polio Information System^§^ and the Global Polio Laboratory Network (GPLN).^¶^ Genomic sequencing and analyses were conducted by WHO-accredited sequencing laboratories within GPLN. A cVDPV outbreak is confirmed when two or more independent detections of genetically linked VDPV emergences are identified through acute flaccid paralysis (AFP) surveillance, environmental surveillance (ES),** or from sampling of healthy community members^††^ (*2*,*3*). Each unique VDPV emergence group is labeled by the country or area (country) and geographic subnational region of the emergence and the number of emergences in each subnational region.

Data on outbreak control were also reviewed. Based on WHO’s Emergency Committee under the International Health Regulations on the international spread of poliovirus, outbreaks were considered to have been interrupted when no detections were identified ≥13 months since the onset of paralysis or collection of the most recent positive environmental or other sample. Outbreaks were considered to be prolonged when transmission persisted for ≥12 months.

### Analysis

VDPV outbreaks were tabulated and mapped by country, serotype, source of detection, emergence group, and other characteristics. cVDPV emergences with ongoing transmission detected outside of the country of first isolation during 2024, either through AFP cases or ES, were also plotted. Descriptive analyses were conducted using R software (version 4.4.1; R Foundation). These activities were reviewed by CDC, deemed not research, and were conducted consistent with applicable federal law and CDC policy.^§§^

## Results

### cVDPV Outbreaks

**FIGURE 1:**
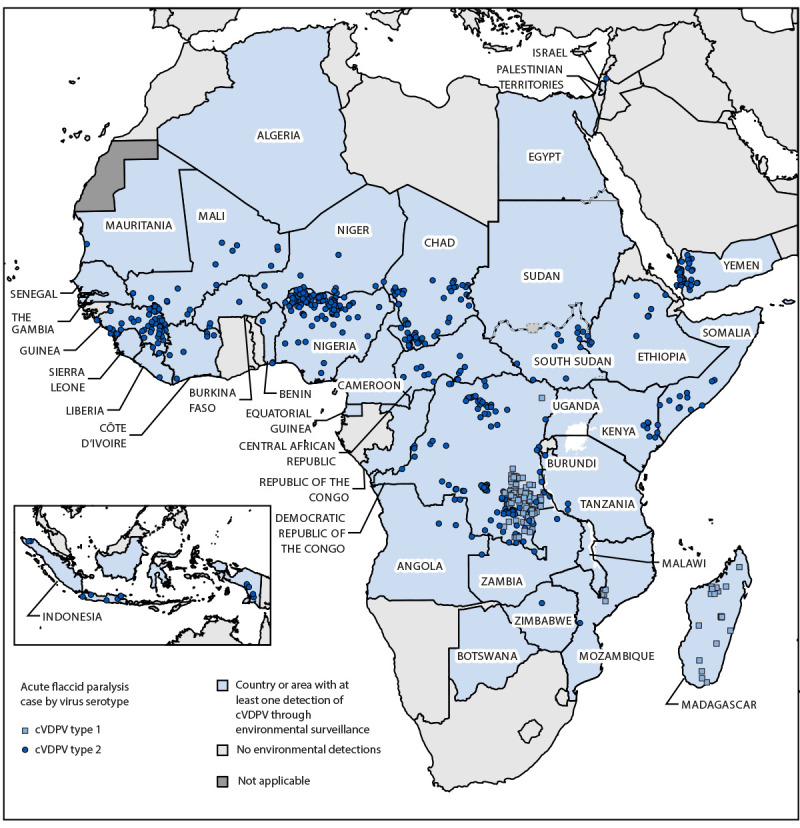
Countries and areas* reporting circulating vaccine-derived polio outbreaks (N = 39) — worldwide, January 2023–June 2024^†^ **Abbreviation:** cVDPV = circulating vaccine-derived poliovirus. * Some boundaries might differ under World Health Organization mapping guidelines. ^†^ Data as of September 18, 2024.

During January 2023–June 2024, a total of 74 cVDPV outbreaks were detected in 39 countries, with 672 confirmed AFP cases identified in 27 of the 39 countries (Figure 1) (Table). Twelve countries reported cVDPV circulation detected only through ES or sampling of healthy community members. Cocirculation of cVDPV1 and cVDPV2 was detected in two countries in the WHO African Region (Democratic Republic of the Congo [DRC] and Mozambique). During the reporting period, no new cVDPV3 emergences were detected. Overall, the number of cVDPV AFP cases declined from 881 in 2022 to 672 during January 2023–June 2024 (Supplementary Figure, https://stacks.cdc.gov/view/cdc/164302). Despite the decline in AFP case counts, the number of countries reporting AFP cases remained approximately the same. The number of cVDPV1 AFP cases declined substantially over the reporting period.

**TABLE:** Ongoing circulating vaccine-derived poliovirus outbreaks (N = 74), by serotype, emergence group, detection source, and other selected characteristics — worldwide, January 2023–June 2024

WHO region/ Country or area	cVDPV emergence designation^†^	Virus origin^§^	Years detected^¶^	No. of detections by source*			% VP1 genome region divergence from Sabin-strain poliovirus^††^	Outbreak confirmation date	Most recent positive sample^§§^
				AFP cases	Other human sources (non-AFP)**	ES			
**cVDPV type 1 outbreaks**									
**African Region**									
DRC^¶¶^	RDC-TAN-1	Sabin	2022–2024	111	0	0	15–27	Sep 12, 2022	Apr 27, 2024
Madagascar	MAD-ANO-2	Sabin	2021–2023	24	1	100	45–65	Feb 28, 2022	Sep 16, 2023
	MAD-SUE-1	Sabin	2020–2023	0	6	0	44–54	Apr 26, 2021	Jun 20, 2023
Mozambique^¶¶^	MOZ-NPL-2	Sabin	2020–2024	5	0	0	47–54	Jul 25, 2022	May 17, 2024
**cVDPV type 2 outbreaks**									
**African Region**									
Algeria	NIE-ZAS-1	Sabin	2022–2024	0	3	30	34–49	Jul 11, 2022	Feb 27, 2024
Angola	RDC-KOR-1	Novel	2023–2024	3	0	7	18–24	Jan 29, 2024	Jul 7, 2024
	RDC-SKV-1	Novel	2024	1	0	0	20–20	Jul 1, 2024	May 11, 2024
	ANG-LNO-3	Novel	2024	1	1	1	13–15	May 6, 2024	Mar 30, 2024
	NIE-ZAS-1	Sabin	2024	0	0	1	31–31	Mar 11, 2024	Jan 24, 2024
Benin	NIE-ZAS-1	Sabin	2022–2024	4	0	4	37–54	Jun 27, 2022	May 18, 2024
Botswana	BOT-FRA-1	Novel	2023	0	0	3	7–10	Aug 14, 2023	Jul 25, 2023
	RDC-MAN-5	Sabin	2022–2023	0	0	2	17–28	Oct 31, 2022	Jan 24, 2023
Burkina Faso	NIE-ZAS-1	Sabin	2023	3	0	1	38–51	Jul 3, 2023	Dec 12, 2023
Burundi	RDC-SKV-1	Novel	2022–2023	1	0	13	6–14	Mar 13, 2023	Jun 15, 2023
Cameroon	CAE-EST-1	Novel	2024	1	0	0	8–8	Sep 10, 2024	May 15, 2024
	NIE-ZAS-1	Sabin	2021–2023	0	0	12	38–48	Oct 25, 2021	Sep 28, 2023
	CAE-EXT-1	Novel	2023	0	0	1	6–6	Oct 9, 2023	Jun 13, 2023
Central African Republic	CAF-BNG-3	Novel	2023	5	2	1	10–14	Jul 10, 2023	Oct 7, 2023
	NIE-ZAS-1	Sabin	2021–2023	6	10	0	36–51	Nov 29, 2021	Sep 9, 2023
	CAF-MOZ-1	Sabin	2023	3	2	0	16–17	Mar 27, 2023	Apr 26, 2023
	CAF-KEM-1	Novel	2022–2023	0	1	0	7–7	May 29, 2023	Mar 9, 2023
Chad	NIE-ZAS-1	Sabin	2021–2024	61	6	7	31–54	Jan 31, 2022	Jun 29, 2024
	CAE-EXT-1	Novel	2023	1	0	0	7–7	Oct 2, 2023	Jul 26, 2023
Côte d’Ivoire	NIE-ZAS-1	Sabin	2022–2024	6	16	68	24–61	Mar 7, 2022	Apr 23, 2024
	RDC-SKV-1	Novel	2023	0	0	1	13–13	Jan 22, 2024	Dec 5, 2023
DRC^¶¶^	RDC-KOR-1	Novel	2023–2024	49	8	34	8–25	Apr 10, 2023	Jun 12, 2024
	RDC-TSH-2	Novel	2024	2	1	0	12–14	Jul 8, 2024	Apr 7, 2024
	RDC-BUE-1	Sabin	2022–2024	11	0	0	24–37	Sep 5, 2022	Mar 20, 2024
	RDC-TSH-1	Sabin	2022–2023	13	0	0	19–36	Oct 3, 2022	Nov 19, 2023
	RDC-HKA-2	Novel	2023	5	0	2	7–16	Jul 17, 2023	Oct 27, 2023
	CAF-BNG-2	Sabin	2023	2	0	0	21–28	Nov 27, 2023	Sep 5, 2023
	RDC-MAN-3	Sabin	2021–2023	32	1	3	23–38	Dec 20, 2021	Aug 4, 2023
	RDC-SKV-1	Novel	2022–2023	14	0	0	6–14	Jan 13, 2023	Jun 23, 2023
	RDC-MAN-5	Sabin	2021–2023	1	1	0	24–24	Mar 14, 2022	Apr 28, 2023
Equatorial Guinea	NIE-ZAS-1	Sabin	2024	0	0	1	38–38	May 27, 2024	Mar 26, 2024
Ethiopia	ETH-TIG-1	Novel	2023–2024	7	0	1	9–22	May 20, 2024	Jun 13, 2024
	RSS-UNL-1	Sabin	2024	5	2	0	12–15	May 20, 2024	May 19, 2024
The Gambia	NIE-ZAS-1	Sabin	2024	0	0	1	45–45	Jun 3, 2024	Feb 15, 2024
Guinea	NIE-ZAS-1	Sabin	2021–2024	52	11	33	40–51	Dec 13, 2021	Jun 12, 2024
Kenya	SOM-BAN-1	Sabin	2018–2024	8	8	9	65–80	Apr 16, 2018	Jun 12, 2024
Liberia	NIE-ZAS-1	Sabin	2023–2024	1	12	18	42–53	Feb 26, 2024	Jun 19, 2024
Malawi	RDC-MAN-5	Sabin	2023	0	0	1	18–18	Feb 27, 2023	Jan 2, 2023
Mali	NIE-ZAS-1	Sabin	2022–2024	16	0	6	37–48	Feb 6, 2023	Jan 2, 2024
Mauritania	NIE-ZAS-1	Sabin	2023	1	0	3	42–47	Dec 4, 2023	Dec 13, 2023
Mozambique^¶¶^	RDC-KOR-1	Novel	2024	0	0	1	19–19	May 6, 2024	Mar 5, 2024
	MOZ-MAN-1	Novel	2023	1	3	0	10–15	Jan 8, 2024	Dec 8, 2023
Niger	NIE-ZAS-1	Sabin	2021–2024	7	0	10	35–66	Nov 1, 2021	Jun 17, 2024
Nigeria	NIE-ZAS-1	Sabin	2020–2024	121	55	100	35–58	Sep 18, 2020	Jun 26, 2024
	NIE-KTS-1	Novel	2023–2024	7	8	1	12–22	Jan 15, 2024	Jun 10, 2024
Republic of the Congo	RDC-KOR-1	Novel	2023	0	0	1	20–20	Jan 22, 2024	Dec 7, 2023
	RDC-MAN-3	Sabin	2023	0	0	1	27–27	Jul 17, 2023	Apr 11, 2023
Senegal	NIE-ZAS-1	Sabin	2023–2024	0	0	6	42–61	Jan 29, 2024	May 2, 2024
Sierra Leone	NIE-ZAS-1	Sabin	2024	0	0	15	43–52	Mar 11, 2024	May 28, 2024
South Sudan	RSS-JON-1	Novel	2024	1	0	3	8–9	Jun 17, 2024	Jun 25, 2024
	RSS-UNL-1	Sabin	2023–2024	6	4	0	13–16	Mar 25, 2024	Jun 28, 2024
	RSS-WEQ-1	Novel	2023–2024	4	3	0	12–18	Jan 1, 2024	Feb 23, 2024
Uganda	SOM-BAN-1	Sabin	2024	0	0	1	68–68	Jun 3, 2024	May 7, 2024
Tanzania	RDC-SKV-1	Novel	2023	2	6	6	13–22	Jul 17, 2023	Nov 20, 2023
Zambia	RDC-SKV-1	Novel	2023	1	4	2	7–13	Jun 5, 2023	Jun 6, 2023
Zimbabwe	ZIM-HRE-2	Novel	2024	0	0	2	7 – 8	Sept 12, 2024	Jun 25, 2024
	ZIM-HRE-1	Novel	2023–2024	1	2	25	8–17	Oct 23, 2023	May 28, 2024
**Eastern Mediterranean Region**									
Egypt	SUD-RED-1	Sabin	2024	0	0	1	12–12	Feb 19, 2024	Jan 31, 2024
	EGY-NOR-1	Novel	2023	0	0	11	9–15	Sep 18, 2023	Dec 30, 2023
Palestinian Territories	EGY-NOR-1	Novel	2024	0	0	6	13–18	Jul 29, 2024	Jun 23, 2024
Somalia	SOM-BAN-1	Sabin	2017–2024	8	6	11	63–77	Feb 12, 2018	Jun 5, 2024
	SOM-BAY-1	Sabin	2023–2024	3	0	1	16–23	Mar 4, 2024	May 14, 2024
Sudan	RSS-UNL-1	Sabin	2024	0	0	1	12–12	Sep 2, 2024	Jan 24, 2024
	SUD-RED-1	Sabin	2023–2024	0	0	6	6–11	Jan 22, 2024	Jan 11, 2024
Yemen	YEM-TAI-1	Sabin	2021–2024	35	2	22	20–45	Nov 22, 2021	May 19, 2024
	SUD-RED-1	Sabin	2023–2024	6	0	1	7–19	Mar 11, 2024	May 13, 2024
**European Region**									
Israel	IUUC-2022	Sabin	2022–2023	1	0	0	12–12	Mar 6, 2023	Feb 13, 2023
**South-East Asia Region**									
Indonesia	cVDPV2	Sabin	2024	1	1	0	13–42	Sep 2, 2024	Jun 15, 2024
	INO-cVDPV2	Novel	2024	6	12	0	8–15	Jul 1, 2024	Jun 27, 2024
	INO-ACE-1	Sabin	2022–2023	6	7	1	27–43	Nov 28, 2022	Dec 7, 2023

**Abbreviations:** AFP = acute flaccid paralysis; cVDPV = circulating vaccine-derived poliovirus; DRC = Democratic Republic of the Congo; ES = environmental surveillance; VDPV = vaccine-derived poliovirus; VP1 = viral protein 1; WHO = World Health Organization.

* During January 2023–June 2024 with data as of September 18, 2024. For AFP cases, the number of patients with a VDPV-positive specimen or for whom a direct contact had a VDPV-positive specimen when the patient did not. For other human sources, the number of contacts of the patient or healthy children in the community with a VDPV-positive specimen. For detections from ES, the total number of samples with VDPVs detected from environmental (sewage) collections.

^†^ Emergences indicate detection of cVDPV strains that have unique genetic reversion compared with other VDPVs, and the names of emergences generally designate the country and geographic subnational region of the emergence’s first detection and the number of emergences in each subnational region.

^§^ Novel refers to the virus strain contained in novel oral poliovirus type 2, a more genetically stable vaccine available since 2021, with lower risk for reversion to neurovirulence than Sabin-strain oral poliovirus type 2.

^¶^ Range of total years detected for previously reported cVDPV outbreaks. Data as of September 18, 2024.

** Contacts and healthy child specimen sampling during January 2023–June 2023 with data as of September 18, 2024, for all emergences.

^††^ Percentage of divergence is estimated from the number of nucleotide differences in the genome region encoding VP1 from the corresponding parental Sabin strain.

^§§^ For AFP cases, dates refer to the date of paralysis onset. For contacts, healthy children, and environmental (sewage) samples, dates refer to the date of collection during January 2023–June 2024 with data as of September 18, 2024. Table is restricted to outbreaks with at least one reported detection during the current reporting period.

^¶¶^ cVDPV type 1 and cVDPV type 2 co-circulated in these countries.

### cVDPV1 Outbreaks

During January 2023–June 2024, cVDPV1 circulation was detected in three countries (DRC, Madagascar, and Mozambique) from four cVDPV1 emergences (Figure 1) (Table). No new countries or emergences were reported since 2022. A total of 140 AFP cases were confirmed, with 111 reported from DRC, 106 (75%) in 2023, and five (5%) in the first half of 2024. Recent cVDPV1 detections occurred in April 2024 in DRC (RDC-TAN-1) and May 2024 in Mozambique (MOZ-NPL-2). The latest cVDPV1 detection in Madagascar occurred in September 2023.

### cVDPV2 Outbreaks

**FIGURE 2:**
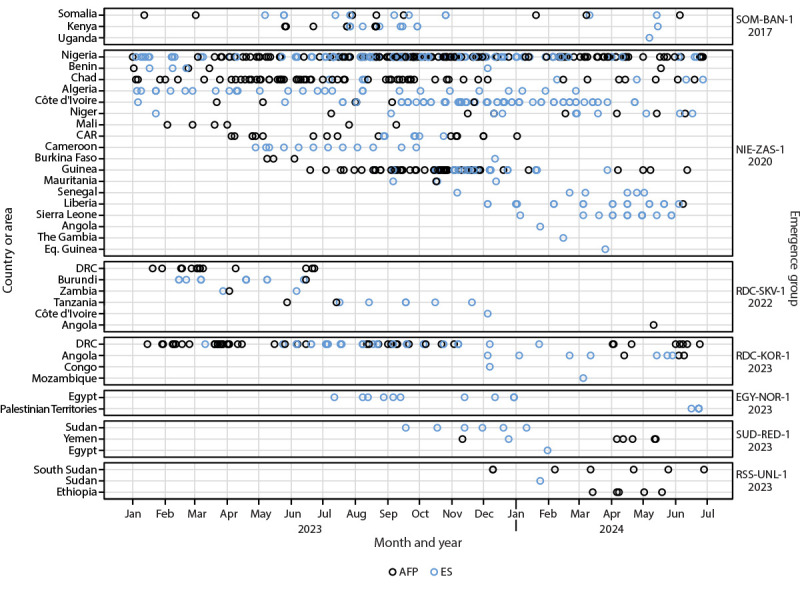
Circulating type 2 vaccine-derived polioviruses* associated with outbreaks ongoing in 2024 that involved international spread since emergence, by outbreak and country or area — worldwide, January 2023–June 2024^†^ **Abbreviations**: AFP = acute flaccid paralysis; CAR = Central African Republic; DRC = Democratic Republic of the Congo; ES = environmental surveillance; Eq. Guinea = Equatorial Guinea. * No international spread was reported from emergence groups in circulating vaccine-derived poliovirus type 1. ^†^ For AFP cases, dates refer to the date of paralysis onset. For environmental surveillance samples, dates refer to the date of collection. For samples collected on the same dates, symbols will overlap; thus, not all isolates are visible. Data as of September 18, 2024, for all emergences.

During January 2023–June 2024, a total of 70 cVDPV2 outbreaks from 34 emergences were reported in 38 countries (Figure 1) (Table); 532 AFP cases were confirmed in 26 countries. During the reporting period, five countries reported their first cVDPV2 detection since type 2-containing OPV was removed from OPV-using countries’ routine immunization programs in April 2016. Ten (29%) of the 34 emergences spread outside the country of first detection. In Nigeria and Somalia, countries with security-compromised areas, persistent cVDPV2 transmission has spread frequently to neighboring countries. The transmission from Nigeria to its neighbors led to further international spread. The NIE-ZAS-1 emergence, first detected in Nigeria in July 2020, continued to circulate within Nigeria during the reporting period and was detected in 17 other countries in the African Region, particularly in West Africa (Figure 2). SOM-BAN-1, detected in Somalia in October 2017 (*7*), was reported in Kenya, and for the first time, was detected in Uganda during the reporting period. Since the previous reporting period (January 2021–December 2022) (*7*), Indonesia has detected two additional cVDPV2 emergences, with seven AFP cases. The EGY-NOR-1 emergence detected in 11 ES samples collected in Egypt was detected in six ES samples collected in the Palestinian Territories in June 2024 (Table).

Among the 70 cVDPV2 outbreaks, 29 (comprising 19 VDPV2 emergences) were linked to nOPV2 use in 19 countries. Two of the 19 emergences (RDC-SKV-1 and CAF-KEM-1) were first detected in September 2022 and December 2022, respectively. A total of 113 AFP cases were reported in 14 of the 19 countries with emergences; the highest number (70; 62%) was reported in DRC. Five countries detected outbreaks linked to nOPV2 use through ES only, with no confirmed AFP cases. The RDC-KOR-1 emergence was first detected in DRC in January 2023 and spread to Angola, Mozambique, and Republic of the Congo. The RDC-SKV-1 emergence was first detected in DRC and spread to Angola, Burundi, Côte d’Ivoire, Tanzania, and Zambia, with the most recent detection of an AFP case in Angola on May 11, 2024.

### Outbreak Control

Of the 74 cVDPV outbreaks, 47 were new outbreaks detected during the reporting period in 30 of the 39 countries reporting outbreaks (Table). The remaining 27 outbreaks had been detected before the reporting period began and are ongoing (<13 months since most recent case) in 20 of the 39 countries. During January 2023–June 2024, SIAs were conducted to control cVDPV outbreaks in 32 of the 39 outbreak countries. Among the 74 cVDPV outbreaks, 11 (in seven countries) were documented to have been interrupted. Prolonged transmission of a cVDPV outbreak (≥12 months from first to most recent detection) was observed in 15 countries: Algeria, Benin, Cameroon, Central African Republic, Chad, Côte d’Ivoire, Democratic Republic of the Congo, Indonesia, Madagascar, Mali, Mozambique, Niger, Nigeria, Somalia, and Yemen. In seven of the 74 outbreaks across six countries, no cVDPV was detected ≥13 months since the most recent positive sample.

## Discussion

Continuing cVDPV outbreaks impede attainment of the Global Polio Eradication Initiative (GPEI) 2022–2026 Strategic Plan goal of eradicating polio, particularly that of interrupting all cVDPV transmission in 2024 (*2*). During January 2023–June 2024, cVDPV2 outbreaks remained as prevalent as those during previous reporting periods (*7*,*8*). Although the number of countries reporting outbreaks is approximately the same during each of the most recent years, as some countries interrupt transmission, newly infected or reinfected countries are reporting confirmed outbreaks.

Since 2022, no new countries have reported cVDPV1 emergences or outbreaks. Although cVDPV1 detections were reported in Mozambique and DRC in early 2024, no detections have been reported in Madagascar since September 2023, following multiyear transmission in each of these countries. This development reflects the ultimate success of outbreak response efforts and highlights the possibility of controlling all cVDPV1 outbreaks in 2024. The decline in routine childhood immunization coverage during the early years of the COVID-19 pandemic (*9*) has resulted in an accumulation of undervaccinated, susceptible children in many African countries with weak essential health services, increasing the risk for new cVDPV1 emergences.

The spread of cVDPV2 emergence groups such as NIE-ZAS-1 and SOM-BAN-1 outside the country of first detection, often with further international spread, reveals gaps in the effectiveness and timeliness of outbreak responses. In light of the many social, economic, and political challenges, promptly interrupting transmission of cVDPV2 requires sufficient resources, including those mobilized within countries, to implement intensive response efforts with cross-border collaborations.

Low supplies of nOPV2, compounded with logistical challenges and insufficient access, have led to delays in implementation of outbreak responses, impeding efforts to achieve the high population immunity required to stop cVDPV2 transmission. During January 2023–June 2024, cVDPV2 outbreaks were linked to nOPV2 use in 19 countries. Whereas nOPV2 is genetically more stable than Sabin strain OPV2 in community circulation, these findings highlight that cVDPVs can develop with nOPV2 use when the timing and quality of vaccination responses are suboptimal (*10*). Prolonged community circulation of the vaccine strain leads to reversion to neurovirulence, seeding new emergences.

Gaps in poliovirus surveillance can delay outbreak response activities and provide a longer opportunity for virus to spread. Efforts are underway to strengthen surveillance systems and improve the capacity to confirm cVDPV outbreaks by increasing the number of laboratories accredited by GPLN to perform genomic sequencing.

The current GPEI target is to stop all cVDPV transmission by the end of 2026. Continued circulation of cVDPVs highlights the need for 1) increased urgency to implement prompt, high-quality SIAs upon detection of new cVDPV outbreaks and 2) enhanced efforts, such as more engagement with humanitarian nongovernmental organizations, to vaccinate children in security-compromised areas and in hard-to-reach communities.

### Limitations

The findings in this report are subject to at least two limitations. First, existing gaps in polio surveillance systems might lead to the underestimation of cases and transmission levels and inaccuracies in the geographic spread of cVDPVs. Second, delays in the transportation of polio samples and testing by reference laboratories might result in underreporting of cases, outbreaks, and emergences during January–June 2024.

### Implications for Public Health Practice

GPEI currently aims to eradicate polio by 2026; the key challenges are ending transmission in security-compromised areas and hard-to-reach communities and preventing any further international spread. Ending transmission by 2026 will require a focus on implementing intensive efforts to vaccinate children in security-compromised and hard-to-reach communities to achieve the goal of sustained cVDPV2 interruption. Countries can control cVDPV outbreaks with timely allocation of resources to implement prompt, high-quality responses after outbreak confirmation. Stopping all cVDPV transmission requires effectively increasing population immunity by overcoming the barriers to reaching children.

## Footnotes

* By genomic sequence analysis of the region encoding capsid viral protein 1, a poliovirus with >1% divergence from the parent Sabin strain for serotypes 1 and 3, or >0.6% for serotype 2, is classified as a vaccine-derived poliovirus (VDPV). Evidence of circulation (i.e., a cVDPV outbreak) occurs when two or more independent detections of genetically linked VDPVs are identified through acute flaccid paralysis (AFP) surveillance, environmental surveillance (ES), or from healthy community members.

† To meet standard laboratory timeliness indicators for stool specimen processing, laboratories should report ≥80% of poliovirus isolation results ≤14 days of specimen receipt, ≥80% of intratypic differentiation results ≤7 days of isolate receipt, and ≥80% of sequencing results ≤7 days of identifying isolate intratype. Results for all acute flaccid paralysis (AFP) cases with paralysis onset or environmental surveillance (ES) collected through June 2024 that have been isolated, differentiated, sequenced, and reported by September 18, 2024, were included.

§ The WHO Polio Information System is a centralized database integrating case-based AFP and ES, with SIA data from all WHO regions. https://extranet.who.int/polis (Access is limited to members of GPEI partner organizations).

¶ GLPN consists of 144 WHO-accredited polio laboratories using the WHO-recommended procedures for detecting and characterizing polioviruses from stool and sewage samples collected from AFP patients, their contacts, and the environment. https://polioeradication.org/resource-hub/?rh_policy_and_report_types=global-polio-laboratory-network-reports

** ES is the systematic sampling and testing of sewage for the presence of poliovirus.

†† Cases of VDPV among healthy community members may be established either through 1) AFP contact sampling or 2) targeted stool sampling of healthy children. AFP contact sampling can be conducted when an AFP case has inadequate stool specimens for laboratory confirmation of poliovirus; AFP contact sampling is used to provide laboratory evidence of poliovirus in an AFP case. Targeted stool sampling of healthy children can be conducted following a new VDPV isolation when community transmission has not been confirmed to determine if poliovirus is present in the community.

§§ 45 C.F.R. part 46.102(l)(2), 21 C.F.R. part 56; 42 U.S.C. Sect. 241(d); 5 U.S.C. Sect. 552a; 44 U.S.C. Sect. 3501 et seq.
